# An Open-Labeled Randomized Prospective Multi-center Study to Evaluate the Efficacy and Safety of Intra-articular Injection of OSSINEXT™, an Autologous Growth Factor Concentrate (AGFC) Compared to Hyaluronic Acid (HA) in Knee Osteoarthritis

**DOI:** 10.7759/cureus.31058

**Published:** 2022-11-03

**Authors:** Mushtaque A Mastim, Chirag Borana, Vrajesh Shah, Rajesh Dhadiwal, Ravi Malhotra, Brijesh Kidiyoor, Amit Kale, Mugdha Gupta, Manishkumar D Shah, Ranjeet Gutte, Anuka Sharma, Vijay Sharma, Ashima Bhatia

**Affiliations:** 1 Global Clinical Development, Wockhardt, Mumbai, IND; 2 Orthopaedics, Masina Hospital, Mumbai, IND; 3 Orthopaedics, VIROC Hospital, Vadodara, IND; 4 Orthopaedics, Dhadiwal Hospital, Nashik, IND; 5 Orthopaedics, Deep Hospital, Ludhiana, IND; 6 Orthopaedics, Yashoda Hospital, Hyderabad, IND; 7 Orthopaedics, Dr DY Patil Medical College, Hospital And Research Centre, Pune, IND; 8 Business Development Hospitals, Wockhardt Hospitals Ltd, Mumbai, IND; 9 Stem Cell Research, Wockhardt Hospitals Ltd, Mumbai, IND; 10 Global Clinical Development, Wockhardt, Delhi, IND

**Keywords:** vas, ikdc, koos, womac, ossinext™, hyaluronic acid, growth factor, knee osteoarthritis

## Abstract

Background: Osteoarthritis (OA) is known as degenerative arthritis and is the second most common rheumatologic problem with a prevalence of 22%-39% in India. Knee OA (KOA) is a major cause of mobility impairment, particularly among females. Non-surgical treatment options for KOA include intra-articular injections of platelet-rich plasma (PRP) and hyaluronic acid (HA). Most commercially available PRP preparation kits do not remove RBCs and WBCs which are detrimental to the healing effects. Wockhardt Regenerative Pvt. Ltd., Mumbai, India has developed a kit known as Ossinext™ which has an advantage over traditional PRP in that it eliminates RBCs and WBCs. This study was conducted to evaluate the effectiveness and safety of intra-articular injection of Wockhardt’s Ossinext™ an autologous growth factor concentrate (AGFC) versus HA in KOA.

Methods: Male and female patients in the age group between 30 and 75 years with confirmed KOA on radiological assessment with Grades I-III on the Kellgren-Lawrence Grading Scale and with visual analog scale (VAS) pain score of 4 or more (on the numeric rating scale) in spite of taking non-steroidal anti-inflammatory drugs (NSAIDs) since past 2 weeks were considered for study participation. This was an open-labeled study and eligible patients were randomly allocated to AGFC or HA in a 1:1 fashion. Three intra-articular injections were given in the affected knee joint, i.e. at baseline, month 1, and month 2 visits. Patients were evaluated at regular intervals, i.e. at months 5, 8, and 11 for primary and secondary endpoints. The primary efficacy endpoint for this study was change from baseline in WOMAC (Western Ontario and McMaster Universities Osteoarthritis Index) scores at month 11 whereas the secondary efficacy endpoints were change from baseline of VAS pain scale at months 1, 2, 5, 8, and 11 as well as change from baseline of WOMAC, KOOS (Knee and Osteoarthritis Outcome System), and IKDC (International Knee Documentation Committee) scale at month 5, 8, and 11. For analysis a mixed model for repeated measures was used.

Results: Out of the 100 patients who were enrolled, 50 patients each were randomized to AGFC and HA arm. The results were analyzed from 99 patients (49 for AGFC and 50 for HA) who met the criteria for the modified intent to treat (mITT) population. At month 11 on the WOMAC scale, there was greater improvement seen with Ossinext™ compared to HA group which was also statistically significant with p-value of 0.0332. Within the group, there was statistically significant improvement before and after treatment in all scales, i.e. WOMAC, KOOS, IKDC, and VAS at all time points, i.e. months 5, 8, and 11 with a p-value as low as <0.0001. Within the group, the VAS score showed statistically significant improvement even at months 1 and 2 as well. A total of 24 patients reported 37 adverse events (AEs) during the study, most common being pain, pyrexia and swelling but none of the AEs reported during the study were considered as severe in intensity. There were no safety concerns reported.

Conclusions: In conclusion, greater and statistically significant improvement was seen with Ossinext™ in WOMAC scores at month 11 compared to HA. Ossinext™ also showed marked statistically significant improvement from before treatment to after treatment in the WOMAC, KOOS, IKDC, and VAS scales used for the assessment of KOA with a p-value as low as <0.0001. Ossinext™ was also safe and well-tolerated.

## Introduction

Osteoarthritis (OA) is known as degenerative arthritis, which commonly affects the large weight-bearing joints, such as the hips and knees, as well as hands, feet, and spine. It is the second most common rheumatologic problem with a prevalence of 22%-39% in India; prevalence increases dramatically with age and it is more common in women than men [1-3]. OA of the knee is a major cause of mobility impairment, particularly among females, nearly 45% of women over the age of 65 years have symptoms while radiological evidence is found in 70% of those over 65 years [2-4]. The main characteristics of OA are hypertrophy of bone at the margins, loss of articular cartilage, subchondral sclerosis, and a range of biochemical and morphological alterations of the synovial membrane and joint capsule [1]. Late-stage pathological changes of OA include ulceration, softening, focal disintegration of the articular cartilage and synovial inflammation may also occur [2, 5]. Pain, particularly after prolonged activity and weight-bearing are typical clinical symptoms; whereas after inactivity stiffness is experienced. It is probably not a single disease but represents the final end result of various disorders leading to joint failure.

Intra-articular injection, as a minimally invasive therapy, for the treatment of KOA is reported to be effective and safe [6]. Non-surgical treatment options for KOA include intra-articular injections of platelet-rich plasma (PRP) and hyaluronic acid (HA) [7]. HA, which is responsible for the viscoelasticity and lubrication of the knee joint, is generated by chondrocytes, synoviocytes, and fibroblasts [8]. Its concentrations are reduced in osteoarthritic knees and evidence suggests that intra-articular injection of HA, which in 2012 has been recommended by the American College of Rheumatology (ACR) [9], is able to reduce the dosage of analgesics, relieve pain, and improve joint function [10].

A meta-analysis recently conducted concluded that intra-articular PRP injection appeared to be more efficacious than HA injection for the treatment of KOA. The PRP injection was superior to the HA injection in terms of long-term pain relief and function improvement, as well as PRP injection, did not increase the risk of adverse events when compared with the HA injection [11]. PRP is an autologous product derived from a patient’s own blood through the process of gradient density centrifugation. PRP contains various growth factors (GFs) and other bioactive molecules, which may promote tissue healing by regulating regenerated tissue structures and aberrant inflammatory processes [12]. Autologous PRP has been widely used with minimum risk of transmission of infectious diseases and immune reactions [13] and intra-articular injections of leukocyte-poor PRP in patients with mild-to-moderate KOA showed clinically significant functional improvement for at least one year [14].

Platelets were thought to have only hemostatic activity, although, in recent years, scientific research and technology have provided a new perspective on platelets and their functions. Studies suggest that platelets contain an abundance of GFs and cytokines that can affect inflammation, angiogenesis, stem cell migration, and cell proliferation.

To obtain the maximum benefit from GFs, it is usually thought that platelets should be maximally concentrated; however, if WBCs are simultaneously concentrated in the platelet fraction, the positive effects of GFs may be reduced. It is well demonstrated that the presence of white blood cells (WBCs) and red blood cells (RBCs) are detrimental to the healing effects demonstrated by the released GFs. Most commercially available PRP preparation kits do not remove RBCs and WBCs and, therefore, the concept of a kit that would produce an acellular GF solution was conceived.

Wockhardt has developed an autologous growth factor concentrate (AGFC) kit called as Ossinext™ (Wockhardt Regenerative Pvt. Ltd., Mumbai, India) for use in KOA and this is the first of its kind of product in the Indian market. The kit contains a proprietary platelet activator, which has an advantage over traditional PRP in that it eliminates RBCs and neutrophils. RBCs show no therapeutic effects for regeneration and may be more painful when injected. Neutrophils, a type of WBC, have inflammatory components which may increase pain and inflammation post-treatment. The kit output called AGFC is acellular in nature, as the kit separates all the degranulated activated platelets and other cellular components. The kit processes blood in a way where the platelets are activated to release their GFs, but without the high concentrations of blood contamination commonly seen in commercially available PRP-based kits. This study was designed to evaluate the effectiveness and safety of intra-articular injection of Ossinext™, an AGFC versus HA in KOA.

## Materials and methods

This study was conducted in accordance with globally accepted standards of GCP (as defined in the ICH E6 Guideline for GCP), in agreement with the Declaration of Helsinki, and in keeping with local regulations at six centers across India. Ethics Committee approvals were obtained from Shree Institutional Ethics Committee (ECR/1149/Inst/MH/2018), Ethics Committee - Dr. D.Y. Patil Vidyapeeth (ECR/361/Inst/MH/2013/RR-19), Institutional Ethics Committee Yashoda Academy of Medical Education and Research (ECR/49/Inst/AP/2013/RR-19), Anand Institutional Ethics Committee (ECR/725/Inst/GJ/2015/RR-21), Institutional Ethics Committee Deep Hospital (ECR/525/Inst/PB/2014/RR-20), and Institutional Ethics Committee Masina Hospital (ECR/1179/Inst/MH/2019).

In the format of an open-labeled study, 100 patients (75 female and 25 male) with confirmed OA of the knee joint by standardized radiological assessment (i.e., standing anteroposterior and lateral X-ray views) with Grade I, II, or III on Kellgren-Lawrence Grading Scale and visual analog scale (VAS) pain score of four or more (on the numeric rating scale) since past two weeks in spite of taking NSAIDs were recruited from six centers in India. Exclusion criteria were Grade IV on Kellgren-Lawrence Grading Scale, past surgery or fracture on knee joint, knee instability, systemic disorders such as rheumatoid arthritis, uncontrolled diabetes mellitus, past or current malignancy, current use of anticoagulant medications, history of known anemia or recent intra-articular injection of corticosteroids (within 30 days), and prior treatment with HA or any other intra-articular injection treatment in the past six months. Complete details of the study procedure were presented to all the patients and written consent was obtained.

After all applicable screening assessments were performed, patients who met all eligibility criteria were randomly allocated to one of the two treatment arms (AGFC or HA) in a 1:1 fashion. The randomization schedule was generated by an independent statistician using block randomization and using SAS software version 9.4 (SAS Institute Inc., Cary, NC, USA). Since this was an open-labeled study, no blinding procedure was required. Study treatment was injected directly into the knee joint, through a superior lateral approach, one line was drawn from the apex of the patella (the apex of the triangle) to the lateral pole of the patella and another line was drawn from the apex to the medial upper pole of the patella, resulting in an inverted triangle (A). The base of the triangle forms the upper border of the patella. The lateral line of the triangle was then marked at the midpoint, where the needle was inserted and directed intra-articularly into the knee joint. No local anesthetic or steroid was used along with AGFC. Three injections of the study drug (AGFC or HA) were given at an interval of one month i.e., at baseline (Day 0), month 1, and month 2. In case of bilateral OA, only one of the affected knees was used for the study. The study treatment (all three doses) was given in the same affected knee and also all the study evaluations were performed on the same knee.

The AGFC was prepared using the Ossinext™ kit by collecting blood (approx. 4 mL) in the Ossinext™ vacutainer blood collection tube followed by mixing the same by inverting the tubes gently six to eight times. The vacutainer tube was then kept standing for 30 min followed by centrifugation at 3400 rpm for 10 min. After centrifugation, the platelet-derived GFs were collected by inverting the tube and inserting a 5 mL syringe with a needle into the tube so that the tip of the needle is just under the cap of the tube. The collected AGFC was then ready to use.

All patients were followed up on monthly basis till month 2 (as the intra-articular injection of study treatment was given on a monthly basis) and then they were followed up every three months till month 11, i.e. at months 5, 8, and 11. At each of these visits, patients were evaluated for an adverse event (AE), concomitant medications, vital examination, physical examination, and VAS score for pain. Western Ontario and McMaster Universities OA Index (WOMAC), Knee and Osteoarthritis Outcome System (KOOS), and International Knee Documentation Committee (IKDC) assessments were done at baseline and at months 5, 8, and 11 (i.e. three, six, and nine months, respectively, post-study treatment).

All statistical analysis was carried out using the SAS software, version 9.4. The primary efficacy endpoint for this study was the change from baseline in WOMAC scores at month 11. The sample size of the study was estimated to detect the improvement in WOMAC scores in both treatment arms with similar precision. Based on the literature of studies comparing AGFC with HA, the standard deviation of change from baseline at month 11 in the WOMAC score was estimated to be 12. It was expected that the treatment effect in each arm (i.e., change from baseline at month 11) was to be at least five points on the WOMAC scale. With 45 evaluable subjects in each treatment arm, the 95% confidence interval, CI, of the treatment effect at month 11 was to have a width of seven. Assuming a 10% dropout rate, 50 subjects in each treatment arm (a total of 100 subjects) were to be randomized in the study. The secondary efficacy endpoints were change from baseline of VAS pain numeric rating scale at months 1, 2, 5, 8, and 11 as well as change from baseline of WOMAC, KOOS, and IKDC scale at months 5, 8, and 11.

A mixed model for repeated measures was used for this purpose. If the normality assumption was not met, then the Rank analysis of covariance (ANCOVA) non-parametric test was used for the analyses. For this purpose, the data were ordered by the magnitude of absolute change-from-baseline using rank transformation. The ranked data were then analyzed by using the ANCOVA model. All primary and secondary efficacy endpoints were analyzed using modified intent-to-treat (mITT) populations. mITT population included all randomized patients who received at least one intra-articular injection, provided a baseline, and have at least one post-injection efficacy measurement. All subjects who have received at least one intra-articular injection of study medication were considered for safety analysis.

## Results

**Figure 1:**
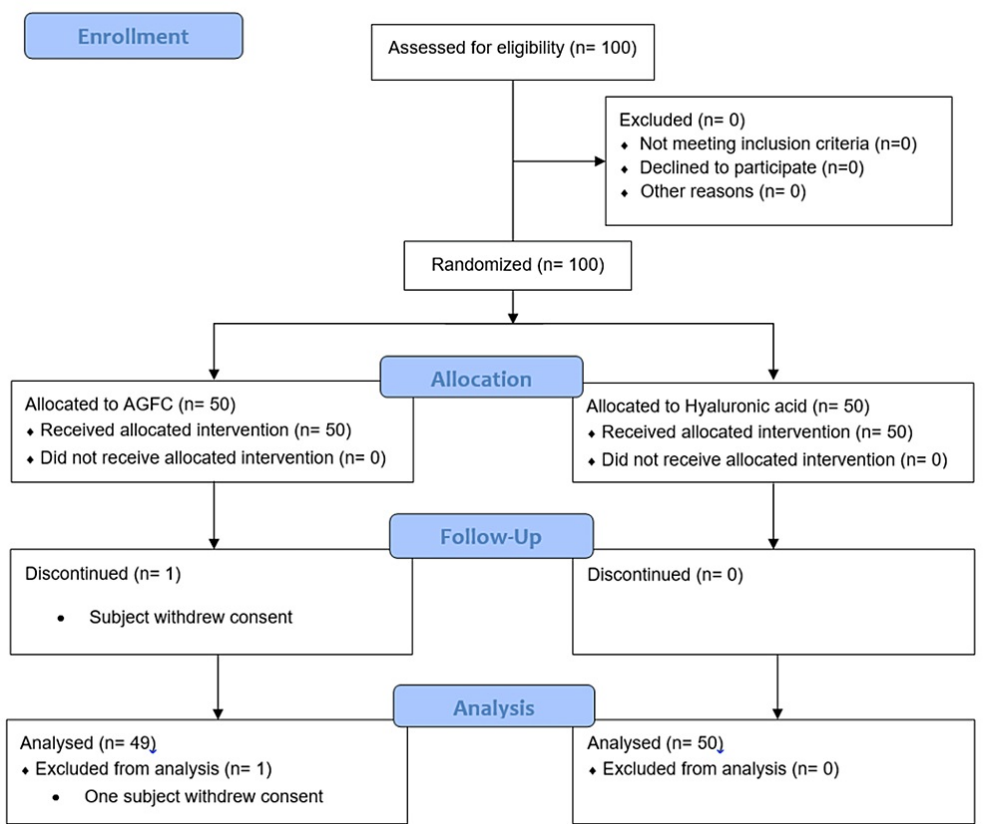
A flow chart of subject enrolment, disposition, and discontinuation.

Out of the 100 patients who were enrolled, 50 patients (38 male and 12 female) were randomized to the AGFC arm and 50 patients (37 male and 13 female) were randomized to the HA arm. Of these, all the patients completed the study, except one subject from the AGFC treatment group who discontinued the study due to voluntary withdrawal before the third dose administration (Figure 1). The median age of the patients from the AGFC treatment group was 52.0 years (range: 30-72 years) and that of patients from the HA treatment group was 53.5 years (range: 30-74 years). All subjects were reported as Indian (from Indian Subcontinent). The median height of the patients from the AGFC treatment group was 157.4 cm (range: 142-176 cm) and that of patients from the HA treatment group was 157.5 cm (range: 137-175.5 cm). The median weight of the patients from the AGFC treatment group was 67.65 kg (range: 42.9-110.1 kg) and that of patients from the HA treatment group was 65.25 kg (range: 42.0-127.2 kg). The median body mass index (BMI) of the patients from the AGFC treatment group was 27.3 (range: 17.3-44.3) and that of patients from the HA treatment group was 26.37 kg (range: 22.4-42.6). All enrolled patients had confirmed OA of the knee joint by standardized radiological assessment (i.e., standing anteroposterior and lateral X-ray views) with Grade I, II, or III on the Kellgren-Lawrence Grading Scale. Distribution of patients based on the Kellgren-Lawrence Grading Scale is as follows -- Grade I (eight patients in the AGFC arm and 12 patients in the HA arm), Grade II (20 patients in the AGFC arm and 17 patients in the HA arm), and Grade III (22 patients in the AGFC arm and 21 patients in the HA arm) (Table 1). Overall, the demographic and baseline characteristics of the patients were comparable between both treatment groups.

**Table 1:** Demographic and baseline characteristics. AGFC, autologous growth factor concentrate; HA, hyaluronic acid; SD, standard deviation; BMI, body mass index

Characteristics	AGFC (N=50)	HA (N=50)
Age (years)		
N	50	50
Mean	53.20	54.66
SD	9.771	10.770
Median	52.50	53.50
Min; Max	(30.00, 72.00)	(30.00,74.00)
Gender		
Female	38 (76.0)	37 (74.0)
Male	12 (24.0)	13 (26.0)
Race		
Indian (from Indian Subcontinent)	50 (100.0)	50 (100.0)
Ethnicity		
Not Hispanic or Latino	50 (100.0)	50 (100.0)
Height (cm)		
N	50	50
Mean	157.8	157.7
SD	6.932	7.844
Median	157.40	157.50
Min; Max	(142.00, 176.00)	(137.00, 175.50)
Weight (kg)		
N	50	50
Mean	68.61	68.29
SD	11.695	13.603
Median	67.65	65.25
Min; Max	(42.9, 110.1)	(42.0, 127.2)
BMI (kg/m2)		
N	50	50
Mean	27.56	27.34
SD	4.528	3.989
Median	27.30	26.37
Min; Max	(17.3, 44.3)	(22.4, 42.6)
Kellgren-Lawrence Grading Scale		
Grade I	8	12
Grade II	20	17
Grade III	22	21

**Figure 2:**
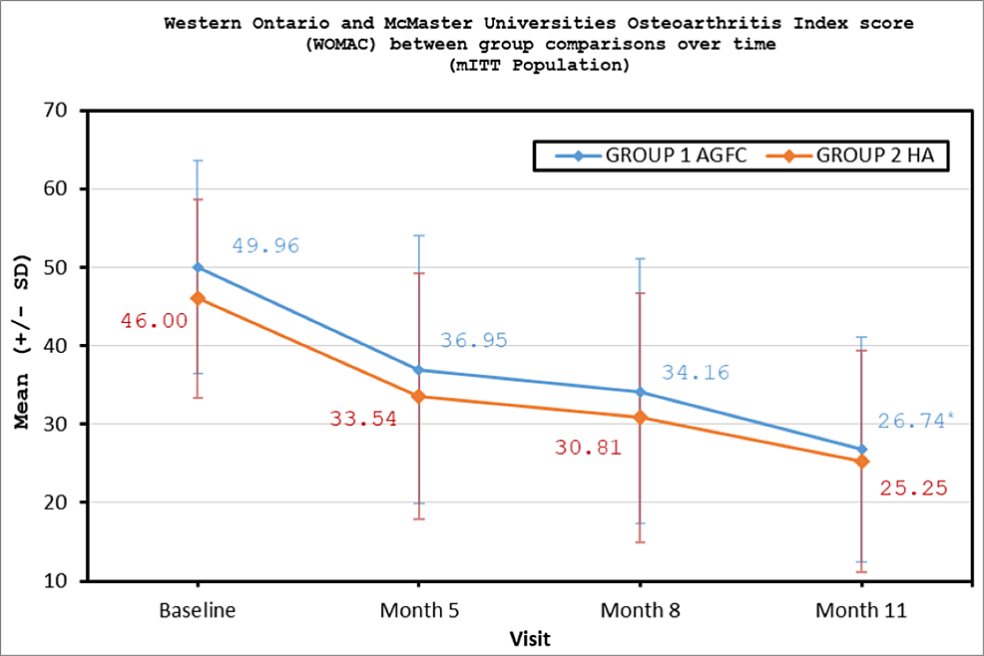
Improvement in WOMAC score between group comparisons over time (mITT population). WOMAC, Western Ontario and McMaster Universities OA Index; mITT, modified intent-to-treat *Statistically significant.

The results were analyzed from 99 patients who met the criteria for the mITT population. Primary endpoint evaluation of the WOMAC score demonstrated a statistically significant change from baseline to month 11 in the mITT population for each of the treatment groups with a p-value of <0.0001. Whereas the rank ANCOVA analysis with the rank of change in WOMAC score as the dependent variable, treatment, and site as fixed effects, and baseline WOMAC score as a covariate was performed (Table 2). The comparison between the treatment groups by rank ANCOVA analysis showed that at month 11 on the WOMAC scale, there was greater improvement seen with Ossinext™ compared to HA groups which were also statistically significant with a p-value of 0.0332 (Table 2, Figure 2). 

**Table 2:** Percentage change in WOMAC score in mITT population. AGFC, autologous growth factor concentrate; HA, hyaluronic acid; SD, standard deviation; CI, confidence interval; mITT, modified intent-to-treat; WOMAC, Western Ontario and McMaster Universities OA Index *Statistically significant

Percentage change from	Statistics	AGFC	HA
Baseline to month 11	N	49	50
	Mean	-23.21	-20.75
	SD	11.486	14.978
	95% CI (within group comparison)	(-27.5, -22.4)	(-25.8, -20.8)
	p-value (within group comparison)	<0.0001^*^	<0.0001^*^
	Covariate p-value	0.0025	
	95% CI [difference between group (AGFC - HA)]	(-43.96, -1.86)	
	p-value [difference between group (AGFC - HA)]	0.0332^*^	

**Figure 3:**
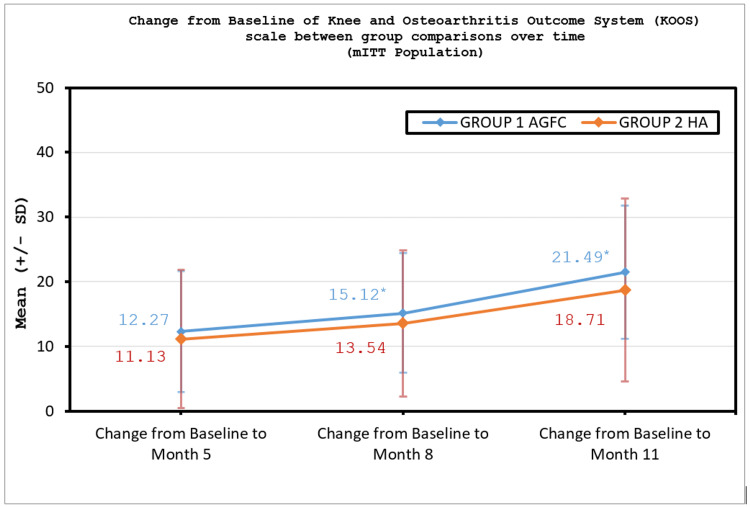
Improvement in KOOS score between group comparisons over time (mITT population). KOOS, Knee and Osteoarthritis Outcome System; mITT, modified intent-to-treat *Statistically significant

Secondary endpoint evaluation of KOOS score by rank ANCOVA analysis in the mITT population at month eight and month 11 demonstrated a statistically significant difference between the treatment groups with a p-value of 0.0363 and 0.0046, respectively (Table 3, Figure 3) whereas the change from baseline to months one, two, five, eight, and 11 in VAS pain numeric rating scale was statistically significant for each of the treatment groups with a p-value of <0.0001 (Table 3), similarly for the other secondary endpoints, i.e. the change from baseline at months five, eight, and 11 in WOMAC, KOOS, and IKDC scale were statistically significant for each of the treatment groups with a p-value of <0.0001 (Table 3).

**Table 3:** Percentage change in VAS, WOMAC, KOOS, and IKDC score in mITT population. VAS, visual analog scale; WOMAC, Western Ontario and McMaster Universities OA Index; KOOS, Knee and Osteoarthritis Outcome System; IKDC, International Knee Documentation Committee; mITT, modified intent-to-treat *Statistically significant

Statistics	N	Mean	SD	p-value (within group comparison)
Percentage change in VAS from baseline to month 1				
AGFC	49	- 0.90	0.549	<0.0001^*^
HA	50	- 0.78	0.526	<0.0001^*^
p-value [difference between group (AGFC - HA)]				0.3306
Percentage change in VAS from baseline to month 2				
AGFC	49	- 2.00	1.295	<0.0001^*^
HA	50	- 1.71	1.139	<0.0001^*^
p-value [difference between group (AGFC - HA)]				0.5171
Percentage change in VAS from baseline to month 5				
AGFC	49	- 2.64	1.440	<0.0001^*^
HA	50	- 2.39	1.461	<0.0001^*^
p-value [difference between group (AGFC - HA)]				0.4210
Percentage change in VAS from baseline to month 8				
AGFC	49	- 2.94	1.405	<0.0001^*^
HA	50	- 2.82	1.606	<0.0001^*^
p-value [difference between group (AGFC - HA)]				0.4766
Percentage Change in VAS from Baseline to Month 11				
AGFC	49	- 3.71	1.335	<0.0001^*^
HA	50	- 3.44	1.609	<0.0001^*^
p-value [difference between group (AGFC - HA)]				0.3073
Percentage change in WOMAC from baseline to month 5				
AGFC	49	- 13.01	10.752	<0.0001^*^
HA	50	- 12.46	11.671	<0.0001^*^
p-value [difference between group (AGFC - HA)]				0.6127
Percentage change in WOMAC from baseline to month 8				
AGFC	49	-15.80	10.382	<0.0001^*^
HA	50	-15.19	12.588	<0.0001^*^
p-value [difference between group (AGFC - HA)]				0.4220
Percentage change in KOOS from baseline to month 5				
AGFC	49	12.27	9.398	<0.0001^*^
HA	50	11.13	10.686	<0.0001^*^
p-value [difference between group (AGFC - HA)]				0.1117
Percentage change in KOOS from baseline to month 8				
AGFC	49	15.12	9.247	<0.0001^*^
HA	50	13.54	11.274	<0.0001^*^
p-value [difference between group (AGFC - HA)]				0.0363^*^
Percentage change in KOOS from baseline to month 11				
AGFC	49	21.49	10.301	<0.0001^*^
HA	50	18.71	14.162	<0.0001^*^
p-value [difference between group (AGFC - HA)]				0.0046^*^
Percentage change in IKDC from baseline to month 5				
AGFC	49	14.26	10.526	<0.0001^*^
HA	50	13.40	11.273	<0.0001^*^
p-value [difference between group (AGFC - HA)]				0.5381
Percentage change in IKDC from baseline to month 8				
AGFC	49	16.96	9.128	<0.0001^*^
HA	50	16.55	12.109	<0.0001^*^
p-value [difference between group (AGFC - HA)]				0.3253
Percentage change in IKDC from baseline to month 11				
AGFC	49	23.32	9.872	<0.0001^*^
HA	50	22.11	14.970	<0.0001^*^
p-value [difference between group (AGFC - HA)]				0.0922

A total of 24 patients reported 37 AEs during the study with 18 patients (36.0%) from the AGFC treatment group and six patients (12.0%) from the HA treatment group reporting at least one AE. There were no deaths, no AEs leading to discontinuation, neither any serious adverse events (SAEs) reported during the study. None of the AEs reported during the study were considered severe in intensity. The most frequently reported AEs were pain, pyrexia, swelling, and hemoglobin decreased. All other AEs were reported as single incidences. Most of the abnormalities observed for laboratory tests, vital sign parameters, and electrocardiogram (ECG) were not clinically meaningful with a few exceptions like two patients (one patient from each treatment group) reported an AE of hemoglobin decreased which were considered as not related to the study drug, three patients (two patients from AGFC treatment group and one patient from HA treatment group) reported with hypothyroidism, one patient from AGFC treatment group reported with blood thyroid stimulating hormone increased all of which were considered as not related to the study drug, one patient from AGFC treatment group reported with events of pyuria and proteinuria which were considered as not related to the study drug and one patient from AGFC treatment group reported with a clinically meaningful ECG abnormality was considered to be due to an event of hypercholesterolemia and was considered as not related to the study treatment. No abnormalities were noted for the physical examinations performed and no pregnancy was reported during the study.

## Discussion

The intra-articular injection is a minimally invasive therapy for the treatment of KOA. Multiple studies have been conducted to evaluate and compare the clinical effect of intra-articular injection PRP and HA in the KOA treatment. Some studies found that patients with PRP injection could not obtain a better clinical outcome than those treated with HA injection [15]. Whereas some studies found that PRP injection was both statistically and clinically superior to HA injection at the end of the 12th month in the treatment of mild-moderate knee OA [16]. A meta-analysis recently conducted concluded that intra-articular PRP injection appeared to be more efficacious than HA injection for the treatment of KOA [8]. PRP injection could provide at least 12 months of pain-free daily living activities but RBCs and neutrophils present in the PRP injections are known to cause painful injections and may increase pain and inflammation post-treatment. The AGFC kit (Ossinext™) developed by Wockhardt for use in KOA contains a platelet activator that eliminates RBCs and neutrophils which have no therapeutic effect for regeneration.

This open-labeled, comparative study was designed to evaluate the safety and effectiveness of Ossinext™ intra-articular injection versus HA in KOA. A total of 100 subjects were enrolled and randomized in a 1:1 ratio to either Ossinext™ or HA treatment arm. A total of 99 subjects completed the study. Overall, the demographic and baseline characteristics of the subjects were comparable between both treatment groups. There were no major protocol deviations reported during the study and all the subjects were 100% compliant with the study treatment administration. The WOMAC score evaluating pain, stiffness, and functional activities of the subjects showed statistically significant improvement from baseline over the period of 11 months on the administration of three doses of Ossinext™ injections. Similarly, the KOOS scale questionnaire related to pain, stiffness, symptoms, functional activities, and quality of life indicated a statistically significant improvement from baseline at each of the timepoints over 11 months. Likewise, the IKDC scale assessing the subjects’ symptoms, daily living, and sports activities too indicated a significant improvement from baseline over time. The VAS pain numeric rating scale score shows a significant reduction in pain from baseline in subjects after administration of Ossinext™ treatment with moderate to minimal pain over the period of 11 months. The rank ANCOVA analysis of the comparison between treatment groups showed Ossinext™ treatment to be statistically significant over HA injection at month 11 by WOMAC score and at month 8 and month 11 by KOOS score. Overall, the treatment with Ossinext™ showed clinically significant functional improvement for nine months after treatment in subjects with mild-to-moderate OA of the knee, and in comparison to the treatment effect with HA, it is clearly visible that after nine months of treatment Ossinext™ shows better and significant improvement in WOMAC and KOOS scales.

The main finding of this study results showed that intra-articular injection of Ossinext™ and HA have an efficacious effect in relieving KOA symptoms. These results depict that within respective groups both Ossinext™ and HA show significant improvement in all the subjective scales used for the study. On comparing the treatment effect between the groups it is clearly visible that after nine months of treatment Ossinext™ shows better and significant improvement in WOMAC and KOOS scales. The long-term effect, i.e. at 12, 18, and 24 months after treatment effect is further being evaluated in the ongoing study. In addition, Ossinext™ injection did not increase the risk of adverse events when compared with HA injection.

There were no deaths, no AEs leading to discontinuation, and neither any SAEs were reported during the study. None of the AEs reported during the study were considered as severe in intensity. Most of the abnormalities observed for laboratory tests, vital sign parameters, and ECG were not clinically meaningful. No abnormalities were noted for the physical examinations performed and no pregnancy was reported during the study.

Our study had a few limitations like it was an open-labeled study, as a result, the investigator, as well as the patient, were aware of the treatment given to them. The long-term effect of the treatment is not presented in this article. In order to evaluate the long-term effect, the study is currently ongoing to evaluate the effect till 24 months post last dose of study treatment.

## Conclusions

In conclusion, at month 11 there was greater improvement seen with Ossinext™ compared to HA groups in the WOMAC scale which were also statistically significant with a p-value of 0.0332, and Ossinext™ injection was better than HA injection in terms of pain relief and function improvement. Ossinext™ also showed marked a statistically significant improvement from before treatment to after treatment in the WOMAC, KOOS, IKDC, and VAS scales used for the assessment of KOA with a p-value as low as <0.0001. Ossinext™ was also safe and well-tolerated.
